# Macrofungal Extracts as a Source of Bioactive Compounds for Cosmetical Anti-Aging Therapy: A Comprehensive Review

**DOI:** 10.3390/nu16162810

**Published:** 2024-08-22

**Authors:** Maja Paterska, Bogusław Czerny, Judyta Cielecka-Piontek

**Affiliations:** 1 Department of Pharmacology and Phytochemistry, Institute of Natural Fibres and Medicinal Plants, Wojska Polskiego 71b, 60-630 Poznan, Poland; maja.paterska@iwnirz.pl; 2 Department of Stem Cells and Regenerative Medicine, Institute of Natural Fibres and Medicinal Plants, 62-064 Plewiska, Poland; boguslaw.czerny@pum.edu.pl; 3 Department of General Pharmacology and Pharmacoeconomics, Pomeranian Medical University in Szczecin, 71-210 Szczecin, Poland; 4 Department of Pharmacognosy and Biomaterials, Faculty of Pharmacy, Poznan University of Medical Sciences, Rokietnicka 3, 60-806 Poznan, Poland

**Keywords:** anti-aging, *Trametes versicolor*, *Schizophyllum commune*, *Tremella fuciformis*, *Pleurotus ostreatus*, *Agaricus subrufescens*, *Volvariella volvacea*, *Ganoderma lucidum*, *Letinula edodes*, *Inonotus obliquus*, mushrooms, macrofungi

## Abstract

For centuries, mushrooms have been used as a component of skincare formulations. Environmental stresses and a modern lifestyle expose the skin to accelerated aging. To slow down this process, natural anti-aging skincare ingredients are being sought. In this review, 52 scientific publications about the effects of chemical compounds extracted from the fruiting bodies of macrofungi on skin cells were selected. The effects of extracts from nine species that are tested for anti-aging effects have been described. According to available literature data, macrofungi contain many polysaccharides, phenolic compounds, polysaccharide peptides, free amino acids, sterols, proteins, glycosides, triterpenes, alkaloids, which can have an anti-aging effect on the skin by acting as antioxidants, photoprotective, skin whitening, moisturizing, anti-inflammatory and stabilizing collagen, elastin and hyaluronic acid levels in the skin.

## 1. Introduction

For centuries, natural ingredients have been sought for use in the treatment of disease or body care. Many biologically active compounds are found in macrofungi. By 2020, 148,000 species of fungi had been described [1], but it is estimated that the actual number is as high as 5 million species, (i.e., more than the number of seed plant species) [2]. Ancient civilizations used mushroom extracts to treat diseases. The Chinese used Reishi and *Tremella* mushrooms for centuries. In ancient Egypt, mushrooms were used to create skincare masks and cleansing preparations [3]. The study of fungi for their chemical composition, particularly in relation to compounds or complexes with beneficial effects on the skin, has gained significant attention in recent years. Fungal species that have been examined are found to contain a variety of biologically active compounds, including polysaccharides, vitamins, peptides and minerals. These compounds can moisturize the skin, inhibit skin aging, relieve inflammation, treat hyperpigmentation and act as photoprotectors. In addition, we found that the levels of phenolic acids and ergosterol in the cosmeceuticals were almost the same as in pure mushroom extracts (85–100%), suggesting no significant loss of bioactivity of these compounds [4]. A cosmetic improves the external appearance of the skin, while a cosmeceutical includes bioactive ingredients that influence the skin’s structure and function at the cellular level. Identifying biologically active compounds with antioxidant properties is a key factor in the development of cosmeceutical formulations. Antioxidants neutralize reactive oxygen species (ROS) but also inhibit tyrosinase and extracellular matrix metalloproteinases, both enzymes involved in skin hyperpigmentation and collagen degradation [5]. Mushrooms are becoming a significant ingredient in cosmetology due to their unusual skincare and health-promoting properties. The increasing use of mushroom extracts in cosmetics and the progressive interest in bio-based chemical compounds with anti-ageing and anti-tyrosinase activity inspired the researchers to prepare this publication. A comprehensive review was conducted, searching specific electronic databases for appropriate articles on the anti-ageing properties of extracts from the fruiting bodies of macroscopic fungi with potential for use in the cosmetic and cosmeceutical sectors. As a result of studies on cellular and animal models, considerable progress has been made in understanding the mechanisms of action of fungal extracts on skin cells. It is essential to evaluate the dermatological potential of new cosmeceuticals by (i) characterising the bioactive properties of compounds that have beneficial effects on the skin; (ii) assessing their penetration through the stratum corneum and the maintenance of effective concentrations over time; and (iii) ensuring efficacy and safety, free from cytotoxicity, irritation or allergic reactions. Given the growing number of studies on the chemical composition of macrofungal extracts and their effects on skin cells, an increase in the use of mushroom-derived compounds in cosmeceuticals is anticipated. This literature review aims to summarise scientific data on the potential anti-aging properties of macrofungus extracts for skincare [6].

## 2. Materials and Methods

**Figure 1:**
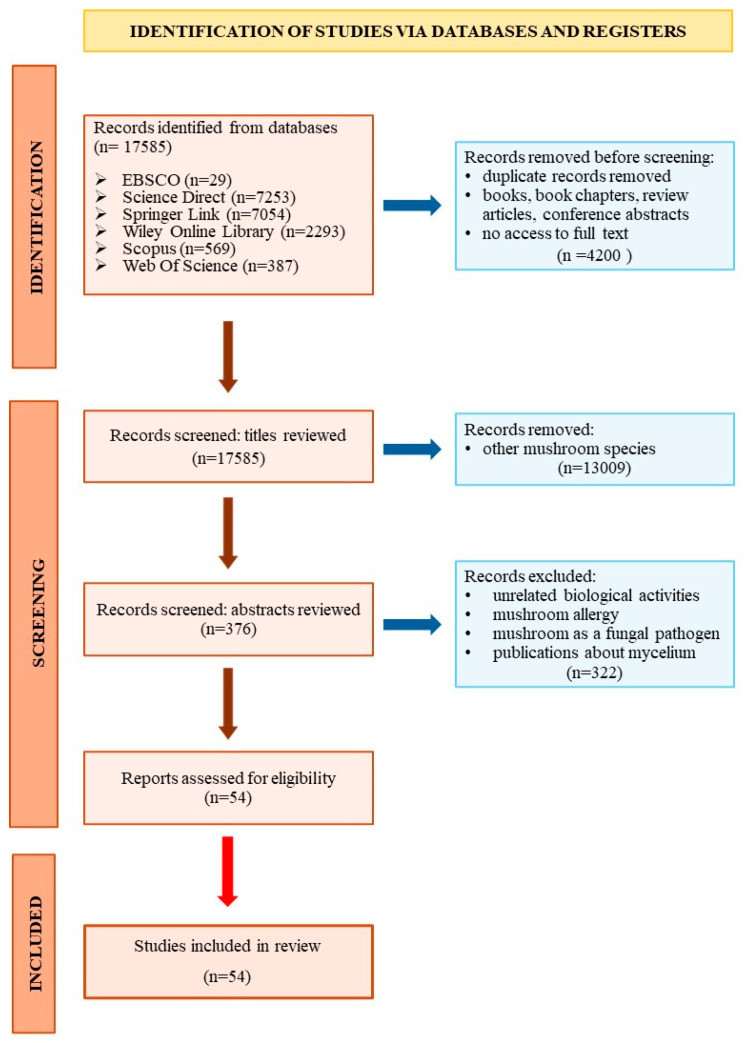
Diagram for literature reviews.

A review was performed of the literature. For available articles, the independent search was conducted in 6 electronic databases (EBSCO, Science Direct, Wiley Online Library, Springer Link, Scopus, Web Of Science) [7]. The search used a keyword combination consisting of the species name and the phrases ‘skin’, ‘fibroblasts’ and ‘keratinocytes’. After entering these keywords, 17,585 results were found. At first, duplicates, books, book chapters, conference abstracts and publications on other mushroom species were excluded. This selection resulted in 376 scientific articles, the abstracts of which were studied. Publications describing mushroom topics other than anti-ageing, publications on infections and allergies caused by mushrooms, and results on using mycelium rather than fruiting bodies were excluded. Only scientific articles in English published in scientific journals were selected. Finally, 52 scientific publications were included in the review (Figure 1).

## 3. Results

### 3.1. The Skin Ageing

**Figure 2:**
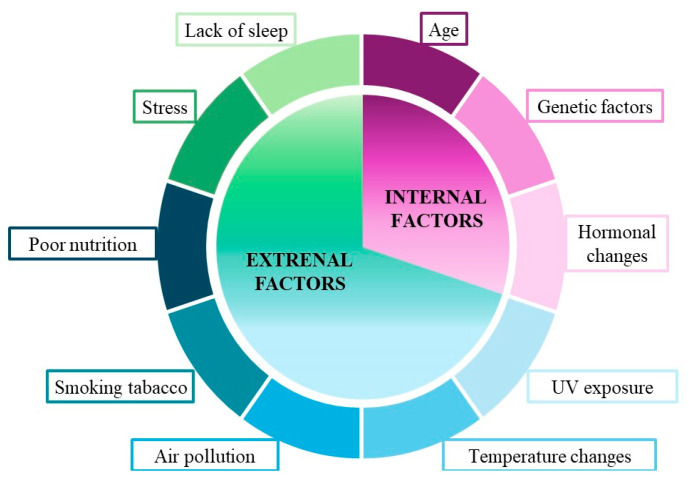
Factors contributing to skin aging.

The skin, as the body’s barrier organ, is continuously exposed to numerous factors that influence its morphology and functions. Skin aging is marked by a gradual decline in functionality and regenerative capacity, making it a multifactorial process. Being the most exposed organ, the skin visibly reflects changes, including aging, which can cause discomfort due to aesthetic imperfections. The aging process of the skin can be categorized into two types: aging driven by endogenous factors and aging influenced by exogenous factors (Figure 2). Endogenous skin aging is related to the age of the body and the physiological changes taking place within it. Endogenous factors contributing to skin aging include the passage of time, genetic predispositions and hormonal balance within the body. This process is closely linked to oxidative stress, characterized by a gradual decline in antioxidant capacity and an increase in the production of reactive oxygen species (ROS).Clinical signs resulting in endogenous skin aging include small wrinkles, dry skin and loss of elasticity. Skin aging caused by exogenous factors is most apparent in areas frequently exposed to the environment, such as the face, neck and hands, and is characterized by deep wrinkles and heightened irregular pigmentation, commonly known as age spots. The main exogenous inducers of skin aging are sunlight (photoaging), air pollution, tobacco smoke, unhealthy eating, exposure to extreme temperatures, stress and lack of sleep [8,9].

**Figure 3:**
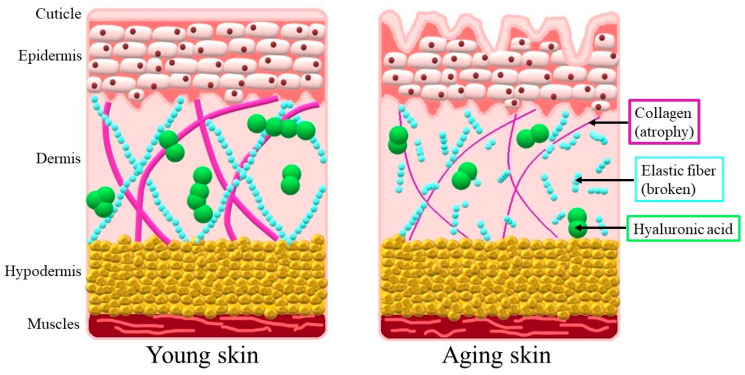
Schematic presentation of the skin morphology.

Aging impacts all layers of the skin, including both the epidermis and the dermis. The aging epidermis shows a reduced ability to perform its barrier (protective) function and is slower to regenerate. The connections between the epidermis and the dermis are gradually degraded, resulting in reduced blood circulation in the epidermis, which consequently leads to less nutrition and oxygenation of the outermost layer of the skin. Skin aging is also connected with reduced levels of hyaluronic acid, changed accumulation of type I and type III collagens, a disruption in the quantitative proportion between them and changes in the organization of elastin fibers (Figure 3). These processes result in the uncomfortable feeling of dry skin, inflammatory changes, loss of skin elasticity causing wrinkles and slower regeneration of skin damage, which causes complications in wound healing. The skin aging process is also manifested by hyperpigmentation disorders [10].

### 3.2. Mushroom Species with Potential Anti-Ageing Effects

#### 3.2.1. *Trametes versicolor* (L.) Lloyd—Yunzhi

It has been shown that extract from the mushroom species *Trametes versicolor* also known as Yunzhi protected against oxidative damage and had antiviral and antibacterial properties. The main bioactive components of the mushrooms of this species are polysaccharide peptides. These compounds have been shown to enhance the activity and gene expression of antioxidant enzymes [11]. Reactive oxygen species (ROS) production is a primary source of cellular damage resulting from UVB radiation exposure. Detailed investigations into the photoprotective properties of extracts from these mushroom species indicate that they may mitigate UVB-induced cellular ageing via a pathway independent of ROS. Therefore, further studies are needed to explain the mechanisms underlying the photoprotective effect of *Trametes versicolor* extracts (10 µg/mL) [12]. In another experiment, polysaccharide peptides (PSPs) were enzymatically hydrolyzed with the enzyme β-1,3-glucanase (80 U/mL), resulting in enzymatic hydrolysates of PSPs extracted from *T. versicolor*. They were proven to exhibit stronger antioxidant activity than PSPs, protecting human skin cells, the keratinocytes, from oxidative damage and having anti-inflammatory activity stronger than PSPs before enzymatic hydrolysis [13].

#### 3.2.2. *Tremella fuciformis* Berk.

Ongoing research is being conducted on the polysaccharides found in the *Tremella fuciformis* mushroom. It has been suggested that these compounds could have various applications in dermatology, including inhibiting the effects of aging, protecting the skin from the sun and speeding up wound healing. In an experiment conducted by Shen et al. [14], human fibroblast extracts containing polysaccharides from the mushroom (0–400 µg/mL) were treated and then exposed to H_2_O_2_. The cell viability assays demonstrated that the polysaccharide exhibited a concentration-dependent protective effect on human skin fibroblasts, with optimal protection observed at a concentration of 200 µg/mL. Treatment with these compounds extracted from *T. fuciformis* resulted in a 51.7% reduction in the production of reactive oxygen species induced by prior H_2_O_2_ treatment, compared to the control. Fibroblasts treated with polysaccharides displayed decreased apoptosis, inhibited activation of pro-apoptotic pathways and enhanced activation of metabolic pathways that support cell viability. Western blot analysis confirmed the downregulating effect of the mushroom polysaccharides, as skin cells showed reduced expression of proapoptotic proteins, compared to cells untreated with the mushroom extract. There was also an increase in the expression of proteins, which have protective functions in DNA stabilization processes and counteract the aging process. These proteins inhibit H_2_O_2_-induced oxidative stress and suppress cell apoptosis. An upregulation of the gene responsible for the synthesis of protective proteins explains the biochemical mechanism of protection of fibroblasts by polysaccharides extracted from *Tremella fuciformis* against oxidative damage.

The effect of polysaccharides on UVA-induced photodamage has been studied in detail in an in vitro study [15,16]. Human fibroblasts were used to experiment. The researchers discovered that polysaccharides application increased skin cell viability. Treatment with polysaccharides diminished the production of reactive oxygen species induced by UVA exposure. Inhibition of lipid oxidation was also observed, while the activity of antioxidant mechanisms was increased. Metabolic pathways that are activated to protect the cell from UVA radiation were analyzed in detail, including the pathway, which is activated by reactive oxygen species emitted as a result of UVA radiation. This pathway promotes antioxidant mechanisms in cells. Polysaccharides application increased the activity of metabolic processes of this pathway, leading to higher expression of genes involved in antioxidant processes. These compounds extracted from the fruiting bodies of *Tremella fuciformis* inhibit UVA damage fibroblasts by activating that metabolic pathway. These results suggest the possibility of using fungal polysaccharides as a photoprotector and have no toxic effects on cells. Other researchers investigated the photoprotective effects of polysaccharides extracted from *T. fuciformis* (5 mg/mL) on human keratinocytes. Their findings demonstrated that these polysaccharides effectively shielded human skin keratinocytes from UV-induced apoptosis and the production of reactive oxygen species (ROS). Additionally, these compounds were shown to upregulate thioredoxin-interacting protein and thioredoxin reductase 2 in primary human keratinocytes, enhancing their photoprotective response. It was also proved that application of polysaccharides can reduce UV-induced skin damage. The test subjects were mice exposed to long-term exposure. Assay results indicated that polysaccharide concentrations ranging from 1 to 5 mg/mL were safe for human skin keratinocytes and significantly enhanced cell viability [17]. The application of polysaccharides extracted from “*Tremella fuciformis*” was found to alleviate oxidative stress in UVA-treated human skin fibroblasts. Treatment with fungal polysaccharides at concentrations between 3.125 and 400 mg/mL led to reduced levels of reactive oxygen species and malondialdehyde in the fibroblasts, while increasing total antioxidant activity. Catalase, superoxide dismutase and glutathione peroxidase activities increased significantly. Polysaccharides extracted from *T. fuciformis* were found to enhance the content of collagen I, hyaluronic acid and elastin in skin fibroblasts treated with UVA. This indicates a potential for these polysaccharides to improve extracellular matrix components in UV-damaged skin cells [16]. Polysaccharides isolated from the fungus *Tremella fuciformis* can also promote wound healing and prevent scars and post-inflammatory hyperpigmentation. A study carried out on human skin cells, fibroblasts and keratinocytes showed that polysaccharides stimulated tissue cell migration, which is the basis of the wound-healing process. Fast wound healing reduces the risk of unaesthetic scarring and post-inflammatory hyperpigmentation. Cells were exposed to varying concentrations of polysaccharides (50, 100, 200 and 300 μg/mL). Significant reductions in melanin content were noted at concentrations of 100, 200 and 300 μg/mL. Specifically, the mushroom extract at 100 and 200 μg/mL effectively diminished melanin production in B16F10 cells and supported wound healing in human HaCaT keratinocytes and Detroit 551 fibroblasts. Furthermore, the treatment did not alter cell morphology or significantly impact the viability of B16F10 cells [15].

Chiang et al. [15] in a next in vitro experiment, using mice cells, discovered that polysaccharides reduced melanin levels. This indicates the potential use of fungal polysaccharides in the prevention and treatment of hyperpigmentation. Bioactive compounds derived from *Tremella fuciformis* could also hold promise for the management of atopic dermatitis and dry skin, conditions commonly associated with aging. In an experiment, fungal polysaccharides (50 and 200 mg/kg) were applied to the mice’s skin. A decrease in the inflammatory response was observed. The results showed that application in both dose reduces pro-inflammatory cytokines levels while increasing levels of regulatory compounds which help regulate an overactive immune system. A decreased level of transepidermal water loss reduced swelling of affected areas, and inhibition of mast cell infiltration was shown. In summary, the application of polysaccharides led to enhanced skin barrier function by reducing transepidermal water loss [18]. A study was performed to assess the safety and effectiveness of using extracts from *Tremella fuciformis* as a moisturizing component in a hand disinfectant. Stable hand sanitizer gel formulations containing 66.5% ethanol and 0.3% triclosan were developed and enriched with snow mushroom polysaccharide extract. Among these formulations, those with 10% snow mushroom extract and 0.3% gelling agent received the highest preference from 20 Thai volunteers. *Tremella fuciformis* extract increased barrier protection and moisturized the skin to a higher degree than disinfectants without fungal polysaccharides. Furthermore, this extract was non-irritating [19]. The ability of polysaccharides to inhibit water loss in the skin, improve barrier function and moisturize the skin shows the potential of this ingredient in cosmeceuticals [20]. An emulsion containing *Tremella fuciformis* extract applied on human skin was tested. The emulsion base consisted of the following ingredients: *Vitis vinifera* (Grape) Seed Oil (25.0%), Glycerin (7.0%), Methyl Glucose Sesquistearate (2.0%), Tocopherol + *Helianthus annuus* Seed Oil (1.0%), Sodium Benzoate + Potassium Sorbate + Aqua (1.0%), Xanthan Gum (1.0%), Sodium Gluconate (0.2%), Citric Acid (for pH regulation), Aqua (to 100.0%). *Tremella fuciformis* extract was added to the emulsion base at a weight concentration of 0.1 wt.%. Dermatological evaluations indicated no swelling or irritation at 48 and 72 h post-application. Treatment with the emulsion containing the mushroom extract resulted in increased epidermal hydration and a 12.4% reduction in transepidermal water loss compared to the control emulsion without the active ingredient. Both microbiological and dermatological assessments, conducted following European Union regulations, confirmed that the applied formulations had no adverse effects on human skin [21]. Chemically modified polysaccharides from *T. fuciformis* were found to be highly effective in mitigating the loss of hydroxyproline and hyaluronic acid in the skin induced by d-galactose. Notably, polysaccharides with a medium molecular weight (4.68 × 10^6^ Da) exhibited superior efficacy in delaying skin aging, reducing oxidative stress and providing anti-inflammatory benefits. The results established a correlation between molecular weight and the in vivo anti-aging activity of the polysaccharides [22].

#### 3.2.3. *Ganoderma lucidum* (Curtis) P. Karst.—Reishi

Extracts of *Ganoderma lucidum* have been employed for centuries in Traditional Chinese Medicine for skincare applications. Currently, research is being conducted on the whitening properties of *Ganoderma* mushroom extracts [23]. The bioactive compounds present in fungal extracts from this species include proteins, glucosides, triterpenoids, alkaloids, phenolic compounds, flavonoids, and polysaccharides. *Ganoderma lucidum* extract (40 μg/mL) demonstrated a 29.34% inhibition of melanin production and a 21.93% reduction in intracellular tyrosinase activity [24]. Similar findings have been reported in numerous studies. Notably, *Ganoderma lucidum* extracts exhibited significant tyrosinase inhibition, with an IC50 value of approximately 0.32 mg/mL, indicating the concentration required to achieve 50% inhibition of enzyme activity. When the reaction mixture contained *Ganoderma* mushroom extracts at a concentration of 1 mg/mL, tyrosinase activity was inhibited by approximately 80%. At a lower concentration of 0.1 mg/mL, the inhibition was around 40% [25]. Furthermore, methanol extracts of *Ganoderma lucidum* inhibited melanin biosynthesis in the B16F7 mouse melanoma cell line. The compound ergosterol peroxide, at a concentration of 2 mg/mL, was identified as the active agent, reducing melanin accumulation by over 1 μg/mL through suppression of the melanogenic enzyme in B16F7 cells. In contrast, ergosterol, a steroid prevalent in mushrooms, did not exhibit a significant effect on melanogenesis in B16F7 cells at the same concentrations [26].

In their study, Vaithanomsat et al. [27] obtained an extract from *G. lucidum* in a concentration 0.5 mg/mL, that contained 40.57% β-glucan and 7.47% protein, with anti-tyrosinase and antioxidant bioactivity. The β-glucan had anti-elastase, anti-hyaluronidase and anti-collagenase activity. These data suggest the applicability of using *G. lucidum* extracts with anti-aging cosmetics [27]. A polysaccharide extracted from *Ganoderma lucidum* at a concentration of 40 μg/mL acts as a natural antioxidant, effectively suppressing UVB-induced photoaging of fibroblasts without causing toxic side effects. Following UVB exposure, there is an upregulation of melanogenesis-related genes—such as microphthalmia-associated transcription factor, tyrosinase, tyrosinase-related proteins 1 and 2, Rab27A and myosin—in both B16F10 mouse melanoma cells and PIG1 human melanocyte cells. However, the polysaccharide from *Ganoderma lucidum* can downregulate the expression of these UVB-induced melanogenesis genes. Additionally, polysaccharides from *Ganoderma lucidum* inhibit the protein kinase A and mitogen-activated protein kinase signaling pathways. These compounds also offer mitochondrial protection from UVB-induced damage, reduce reactive oxygen species production and prevent UVB-induced synthesis of cyclic adenosine monophosphate, contributing to their capacity to inhibit skin photoaging [28].

The findings demonstrated that a polysaccharide extracted from *Ganoderma lucidum* at a concentration of 5 g/L can safeguard human skin fibroblasts from oxidative damage induced by H_2_O_2_. This protective effect is mediated through the upregulation of enzyme activity and the activation of cellular defence mechanisms that modulate the expression of antioxidant and detoxification enzymes in response to oxidative stress and other damaging stimuli. The polysaccharide has antioxidant activity, protecting the skin from oxidative stress damage [29]. In 2022, in an extract from *G. lucidum* a new octapeptide was discovered, which was named PVRSSNCA. At a concentration of 1 mg/mL, the octapeptide demonstrates strong antioxidant and tyrosinase inhibitory activities, influencing various melanogenic enzymes, while exhibiting minimal cytotoxicity to cells. Proteomic analysis has identified the impact of the octapeptide on proteins associated with tyrosinase and melanogenesis. Concentrations ranging from 1.5625 µg/mL to 100 µg/mL of the octapeptide did not result in significant cell death in either melanoma cells or Vero cells [30].

#### 3.2.4. *Lentinula edodes* (Berk.) Pegler—Shiitake

Mushrooms of the species *Lentinula edodes* are abundant in biologically active compounds such as l -ergothioneine and lentinan, which is a polysaccharide ((1-3), (1-6), β-D-glucan), gentamycin, as well as phenolic acids, including protocatechuic acid with potent anti-aging activity [31,32]. A lot of compounds with therapeutic activity have also been isolated, such as terpenes, alkaloids, polysaccharides, steroids, phenolic compounds, glycosides and flavonoids. Cytosine and Adenosine were detected in the ethanol extract obtained from *L. edodes*. Adenosine was evaluated against arbutin, revealing that adenosine exhibited superior activity and could be utilized as an effective skin-whitening agent due to its potential to inhibit the melanogenesis signalling pathway. Ergosterol, and cytosine, were also detected in the *L. edodes*, which also inhibited melanogenesis, suggesting that extracts from the shiitake mushroom may find application in the production of skin-whitening products [32]. L-ergothioneine and lentinan also showed strong antioxidant and anti-inflammatory activity. It suggests potential suitability for use in anti-aging cosmetics [33]. In research by Zi et al. [34], lentinan demonstrated significant antioxidant activity by effectively scavenging free radicals and superoxide anions. The quenching rates for DPPH radicals ranged from 0.094 to 1.5 mg/mL, while for ABTS radicals and superoxide anions, the quenching rates ranged from 23.44 to 375 µg/mL, showing a concentration-dependent effect. In addition, lentinan prevented damage to H_2_O_2_-treated human keratinocytes (HaCaT) cells, reduced malondialdehyde synthesis and increased superoxide dismutase activity. This compound not only protected the cells from oxidative damage but in addition, inducing repair was also observed in HaCaT cells. The lentinan can induce an increase in the tolerance of human keratinocytes cells to oxidative damage, and enhance stress resistance, and protective and regenerative activity was demonstrated. The reported findings suggest that extracts derived from *Lentinula edodes* mushrooms possess potential applications in cosmetology. In subsequent research, the impact of lentinan on skin aging caused by exposure to benzo(a)pyrene was evaluated.

This chemical compound is an environmental pollutant, which accelerates skin aging by inducing oxidative stress and increasing the production of inflammatory mediators. Lentinan was found to mitigate oxidative stress induced by benzo(a)pyrene, as evidenced by a concentration-dependent decrease in reactive oxygen species in human keratinocyte cells. The activities of superoxide dismutase and glutathione peroxidase were approximately 18-fold and 2.7-fold higher, respectively, in cells treated with lentinan compared to controls. Additionally, lentinan significantly lowered both the mRNA and protein levels of interleukin-8 and chemokine-2 ligand. These findings indicate that the compound extracted from *Lentinula edodes* effectively reduces benzo(a)pyrene-induced oxidative stress and inflammation, thereby providing protection to skin cells against detrimental environmental factors [34]. The mushrooms *L. edodes* synthesize trehalose. This phenomenon is known as anhydrobiosis, which refers to the capacity of organisms to endure extended periods of desiccation. Trehalose’s ability to retain moisture renders it valuable as a hydrating agent in cosmetic formulations. This compound is also an antioxidant [35,36]. Shiitake mushroom extract is rich in pantothenic acid and kojic acid, bioactive compounds that inhibit melanogenesis [37]. Shiitake mushroom extracts are active in inhibiting melanin production through tyrosinase inhibition [33,38].

Application of extracts at a concentration of 100 μg/mL reduced pigmentation in zebrafish embryos by almost 1/3 compared to the control. Increasing the concentration to 300 μg/mL stronger reduced pigmentation. This fact confirms that shiitake mushroom extracts contain chemical compounds that effectively inhibit melanin production. The decrease in colouration observed in fish is likely linked to the inhibition of tyrosinase, the primary enzyme involved in enzymatic browning and melanogenesis in both animals and humans [39]. Notably, extracts from *Lentinula edodes* exhibited no toxic effects on zebrafish embryos [39]. These findings indicate the potential for using these extracts in skin-whitening cosmeceutical products.

#### 3.2.5. *Schizophyllum commune* Fr.

*Schizophyllum commune* is rich in phenolic compounds and polysaccharides with notable antioxidant properties, such as schizophyllan, a β-glucan. A strong correlation was found between the total phenolic content of extracts from this species and their ability to scavenge 2,2-diphenyl-1-picrylhydrazyl (DPPH) radicals [40]. Schizophyllan exhibits antioxidant, anti-aging and melanin production inhibitory activities, with an optimal antioxidant effect observed at a concentration of 2.0 mg/mL. Increasing concentrations of schizophyllan in the medium reduced UVB-induced radiation damage, with the highest protective effect seen at 1 mg/mL. At this concentration, schizophyllan pretreatment increased cell viability by 23.5% compared to the control group.

In a related study, the effect of schizophyllan on reactive oxygen species (ROS) production in UVB-exposed skin cells was examined. UVB irradiation significantly elevated fluorescence in cells, indicating increased ROS accumulation. Pretreatment with schizophyllan reduced fluorescence in a concentration-dependent manner, although it did not restore ROS levels to baseline. Nonetheless, schizophyllan significantly mitigated the rise in ROS levels [41].

Further research demonstrated that polysaccharides from *Schizophyllum commune* possess antioxidant activity by scavenging superoxide anions and hydroxyl radicals. Various extracts from the fruiting bodies of this wild basidiomycete, including hot water extract (HWE), hot water polysaccharides (HWP) and hot alkali polysaccharides (HWAE), were evaluated for their antioxidant properties. The total phenol content was highest in HWP and HWE, followed by HWAE. The median effective concentrations (EC50 values) for antioxidant activities were 8.3 ± 0.1, 6.9 ± 0.0 and 8.9 ± 0.1 mg/mL, with DPPH scavenging activities showing values of 0.8 ± 0.0, 0.6 ± 0.0 and 1.8 ± 0.0 mg/mL for HWE, HWP and HWAE, respectively. Reducing power EC50 values were 7.6 ± 0.1, 7.9 ± 0.0 and 12.5 ± 0.1 mg/mL, and ferrous ion chelating abilities were 3.1 ± 0.0, 4.6 ± 0.1 and 4.9 ± 0.1 mg/mL. These EC50 values for antioxidant activity, DPPH scavenging and reducing power were correlated with both total polysaccharide and total phenol content [42].

In the next study, Cheng et al. [43] isolated two types of polysaccharides: intracellular polysaccharides and extracellular polysaccharides. Polysaccharides from both groups can reduce reactive oxygen species, the higher efficiency shown by intracellular form. A dose-dependent relationship between the samples and cell viability was observed. Similarly, the positive control exhibited a range of cell viabilities from 5% to 99.36% at concentrations between 0 and 200 mg/mL. For intracellular forms at concentrations ranging from 0.08 to 10 mg/mL, cell viability varied from 72.19% to 104.82%. When the concentration of the extracellular form was 2.5 mg/mL or lower, cell viability exceeded 80%. Consequently, a concentration of 2.5 mg/mL was selected for both intracellular and extracellular polysaccharides in the follow-up experiments conducted over a 24 h period.

The strong antioxidant and anti-tyrosinase activity of aqueous extracts from *S. commune* (10 mg/mL) was demonstrated in the next experiment. Depending on the extraction time, the free phenolic content ranged from 30.73–41.07 mg Gallic Acid Equivalent per gram extract, while polysaccharide levels ranged from 38.63–43.48 mg Gallic Acid Equivalent per gram extract. Antioxidant activity, as measured by the DPPH assay, ranged from 80–87% depending on the extraction time, while tyrosinase inhibition ranged from 10 to 95%. Because of the results obtained, it has been suggested that *S. commune* extracts could become a valuable cosmeceutical resource to slow down the skin aging process and cure skin pigmentation [44].

Interestingly, chemical compounds extracted from mushrooms can also be used as agents that act indirectly to inhibit signs of skin aging. A conjugate consisting of papain, a proteolytic enzyme and *Schizophyllum commune*-glucan, a high-molecular-weight polysaccharide derived from *Schizophyllum commune*, was utilized. *Schizophyllum commune*-glucan was shown to improve the stability of papain, which is used as an agent to promote drug transdermal permeation. Transdermal permeation of antipyrine was markedly accelerated by a 1 h pretreatment with the *Schizophyllum commune*-glucan-papain conjugate. The cumulative amount of antipyrine permeated over 10 h increased nearly elevenfold compared to the control when the skin was pre-treated with a 2.0% *Schizophyllum commune*-glucan-papain conjugate. This conjugate was found not to cause skin irritation, indicating its potential for effective use in transdermal drug delivery systems.

#### 3.2.6. *Inonotus obliquus* (Ach. ex Pers.) Pilát—Chaga

Extracts from the *Inonotus obliquus* were applied to human fibroblast cells previously treated with H_2_O_2_, which resulted in a reduction of reactive oxygen species levels in the cells and the inhibition of lipid peroxidation. The study indicated that *I. obliquus* extracts protected human fibroblasts from hydrogen peroxide-induced apoptosis and premature cell aging. The intracellular ROS scavenging activity of *Inonotus obliquus* was measured at 33% at a concentration of 25 µg/mL. At this concentration, *Inonotus obliquus* demonstrated a 26% inhibition rate, compared to just 7% inhibition in the untreated control group. Furthermore, *Inonotus obliquus* extract effectively mitigated UV-induced morphological alterations, including skin thickening and wrinkle appearance. It was also reported to increase collagen synthesis by inhibiting matrix metalloproteinases activity in human fibroblasts treated with hydrogen peroxide. In these experiments, hydrogen peroxide treatment reduced the cell viability of the control group to 48.4%. However, treatment with *Inonotus obliquus* at a concentration of 25 µg/mL improved cell viability to 69.5% [45]. Inotodiol isolated from Chaga shows anti-inflammatory activity in human skin fibroblasts. The extract is effective in the treatment of skin inflammation and atopy and has antioxidant properties. The compound has anti-allergic effects, selectively inhibits inflammation in mast cells, and induces the maturation of dendritic cells. The compound exhibits anti-allergic properties, specifically targeting inflammation in mast cells and promoting dendritic cell maturation. Poly (I:C) treatment at concentrations ranging from 0 to 80 μg/mL did not result in cytotoxicity. In contrast, inotodiol demonstrated approximately 70% cytotoxicity at doses of 20 μg/mL or higher [46].

Exposure of human skin cells to UVB radiation led to the overexpression of pro-inflammatory cytokines. However, inotodiol significantly reduced the expression of the genes involved in this inflammatory response. Additionally, inotodiol modulated collagen and hyaluronic acid synthesis by influencing the expression of related genes, thereby mitigating skin aging. Cells treated with various concentrations of inotodiol (0–20 μg/mL) for 24 h showed decreased mRNA expression of cytokines at the lowest concentration (2.5 μg/mL). Inotodiol was non-toxic, with cell viability exceeding 70% at all concentrations except for the highest tested concentration of 20 μg/mL [47].

#### 3.2.7. *Pleurotus ostreatus* (Jacq.) P. Kumm.

The results showed that polysaccharides extracted from *Pleurotus ostreatus* can scavenge DPPH and ABTS free radicals, absorb water and have an inhibitory effect on collagenase and elastase activity. The extract obtained by extraction with 80% ethanol had the highest efficacy. Reactive oxygen species, aging cells, regulators genes immune responses and pro-inflammatory cytokines were shown to increase after UVA irradiation of human fibroblasts. Cells pretreated with polysaccharides isolated from *Pleurotus ostreatus* mushrooms at a concentration of 50 µg/mL exhibited notable reductions in reactive oxygen species levels and the number of aging cells. Additionally, there was a decrease in the activity of genes regulating immune responses, as well as a reduction in the secretion of interleukin-6 and other proteins involved in inflammation and immune responses. The inhibition of reactive oxygen species accumulation and aging cell numbers was comparable between the *P. ostreatus* mushroom extract (extracted with 80% ethanol) and quercetin, which is known for its anti-inflammatory properties. These findings suggest that polysaccharides from *P. ostreatus* may offer protective benefits against photo-aging in human skin cells [48].

Further testing of *P. ostreatus* extracts for skin permeation was conducted ex vivo using a Franz Vertical Diffusion Cell apparatus with pig ear skin as the membrane. The results indicated no toxicity to keratinocytes and fibroblasts in a concentration-dependent manner, supporting the safety of these extracts for use in cosmeceutical applications. Additionally, no permeation of phenolic acids present in *P. ostreatus* extract was detected, affirming the safety of these extracts as skincare ingredients. In tests with HaCaT cells, cell viability remained at up to 90% at 100 μg/mL concentration, while the highest concentration tested (10 mg/mL) significantly reduced cell viability [49].

An experiment was conducted to test the hypothesis that a cream containing a β-glucan extracted from the *P. ostreatus* mushroom—pleuran—would potentially provide relief from sun-induced skin damage caused by UVA/UVB exposure, and evaluate the effectiveness of the cream after 30 days of application to both the face and body. The cream with added mushroom polysaccharides alleviated erythema caused by UV exposure. Moreover, continuous application of the cream over a 30-day period led to enhanced hydration, improved skin brightness and increased elasticity on both the face and body. In summary, these findings suggest that β-glucans are effective active ingredients with UV-protective properties and can alleviate sunburn resulting from excessive UV exposure. Their properties may prevent photo-aging of the skin. The cream composition consisted of aqua (72.0%), glycerin (5.0%), stearic acid (3.0%), *Helianthus annuus* seed oil (7.0%), cetyl alcohol (3.0%), beta-glucan (from *Pleurotus ostreatus*) (2.0%), sorbitan stearate (1.0%), polysorbate 60 (1.0%), phenoxyethanol (1.0%), tocopherol (0.5%), allantoin (0.5%), citric acid (0.5%) and xanthan gum (0.5%) [50]. Pleuran, an insoluble beta-D-glucan in hydrogel form, offers a natural alternative to the commonly used soluble beta-D-glucans derived from chemical sources. When applied to primary cultures of human keratinocytes, pleuran induced the release of matrix metalloproteinase-9 (MMP-9) and matrix metalloproteinase-2 (MMP-2) after 24 h of incubation. A concentration-dependent increase in the release of pro-MMP-9 was observed across a range of 2 to 200 µg/mL, whereas the release of pro-MMP-2 remained unchanged. Furthermore, no active forms of both metalloproteinases were detected, demonstrating that there was no autoactivation of inactive enzyme precursors in vitro. The results indicate that pleuran application stimulates keratinocytes to release the zymogen pro-metalloproteinase-9, which is involved in tissue remodeling and wound healing. The obtained results suggest the use of this polysaccharide in dermatological therapies and the suppression of skin aging [50].

The effect of local dermal application of an aqueous-alcoholic extract of *Pleurotus ostreatus* fruiting bodies (5% and 10% formulated with Vaseline) on wound skin healing in mice was studied. The antimicrobial, anti-inflammatory andantioxidant effects of the extract were demonstrated, as well as its ability to increase collagen synthesis and neovascularization. Increased activity of the protein that regulates the growth, proliferation and differentiation in epidermal cells (epidermal growth factor) has been reported. These findings underscore the potential therapeutic benefits of *Pleurotus ostreatus* extracts for skin regeneration. A maximum concentration of 10% (*w*/*w*) of the mushroom extract was demonstrated to be safe in mice after a 14-day observation period [51]. *Pleurotus ostreatus* is notably rich in free amino acids, which are crucial for skin hydration. Free amino acids, key components of the natural moisturizing factor, play an essential role in maintaining skin moisture levels; their deficiency can lead to dry skin conditions. Thus, this study aimed to analyze the free amino acid content in selected Ethiopian plant and fungal species, including *Pleurotus ostreatus*. The extract of *P. ostreatus* was found to contain a substantial amount of free amino acids, with a concentration of 400.01 mg/g [52].

#### 3.2.8. *Agaricus blazei* Peck (*Agaricus subrufescens*, *Agaricus brasiliensis*)

The impact of ethanol extract on keratinocyte cell viability was evaluated, utilizing this cell line as a validated in vitro model for assessing the safety of cosmeceutical ingredients. The ethanol extract from *Agaricus blazei* demonstrated no cytotoxicity at concentrations up to 100 µg/mL. Nonetheless, a reduction in cell viability of up to 30% was observed at 1 mg/mL, with the most significant decrease occurring at the highest tested concentration of 10 mg/mL. These findings indicate that elevated concentrations of *Agaricus blazei* extract may exhibit toxicity towards skin cells [49]. Experiments were conducted to prove that *A. blazei* mushroom extract inhibited melanin synthesis, increased collagen synthesis and upregulated the expression of hyaluronan-2, 3 and aquaporin-3 synthase genes at a concentration of 100 μg/mL. The chemical composition of extracts was analyzed and the following chemical compounds were identified: ergosterol, 5-dihydroergosterol, cerevisterol, cerebroside B, cerebroside D, adenosine and benzoic acid. Two cerebrosides and three ergosterol derivatives, previously untested for their biological activity, were selected for evaluation. Among these, 5-dihydroergosterol was found to inhibit melanogenesis and promote collagen biosynthesis in human skin fibroblasts. Additionally, cerevisterol, cerebroside B and cerebroside D were shown to block nitric oxide production in cells. Notably, cerebroside D enhanced the expression of hyaluronate synthase-2 and aquaporin-3 genes in keratinocytes [53].

Tyrosinase, a crucial enzyme in melanin synthesis, was the focus of a study assessing the effect of an aqueous extract from *Agaricus blazei* on its activity. Using l-tyrosine and l-3,4-dihydroxyphenylalanine as substrates, the extract exhibited inhibitory effects on tyrosinase activity similar to those of arbutin and vitamin C, in a concentration-dependent manner. Treatment with *Agaricus blazei* fruiting body extracts (ranging from 3–100 µg/mL) reduced melanin content by 57% compared to the control. Additionally, the extract decreased nitric oxide production, which is known to enhance tyrosinase activity in melanocytes [54].

Furthermore, methanolic extracts from *Agaricus brasiliensis* demonstrated an inhibitory effect on melanin synthesis, reduced the expression of melanogenesis-related proteins and lowered intracellular reactive oxygen species levels. The extract inhibited tyrosinase activity (IC50 = 0.713 mg/mL) and reduced melanin production (IC50 = 0.711 mg/mL) in the melanin-producing B16F10 mouse melanoma cell line. This extract also decreased levels of tyrosinase and tyrosinase-related protein 1. The findings suggest that *Agaricus brasiliensis* methanolic extract has potential as a natural skin-whitening agent in skincare products [55].

#### 3.2.9. *Volvariella volvacea* (Bull.) Singer

Polysaccharides derived from *Volvariella volvacea* were evaluated for their potential as multifunctional cosmetic ingredients, assessing both their in vitro and in vivo efficacy. The polysaccharides were extracted using three methods: hot water shaking, microwave-assisted extraction and ultrasound-assisted extraction. The effectiveness of each method was compared based on extraction yield, polysaccharide content and biological activities, including antioxidant, anti-tyrosinase and anti-elastase activities.

Hot water shaking extraction yielded the highest extraction efficiency (15.58 ± 0.96% *w*/*w*) and the greatest beta-glucan content (18.80 ± 0.81% *w*/*w*). Extracts obtained through this method exhibited the most significant inhibitory effects on lipid peroxidation (IC50 = 0.0378 mg/mL), tyrosinase activity (51.46 mg Ketorolac Acid Equivalent per gram) and elastase activity (604.21 ± 73.66 mg Epigallocatechin Gallate per gram).

To ensure safety, the cytotoxicity of the polysaccharides was assessed. A cosmetic cream-gel incorporating these polysaccharides was formulated, and the 0.2% concentration demonstrated the most durable colour retention. The cream’s UV protection factors, skin irritation (assessed via patch tests) and in vivo efficacy—including effects on skin hydration, wrinkle reduction and whitening—were evaluated. The polysaccharides from *Volvariella volvacea* showed no cytotoxicity to human skin fibroblasts. The cream gel containing these polysaccharides improved skin hydration, elasticity and firmness. After 8 weeks of regular application, noticeable reductions in skin roughness, flakiness, wrinkles and melanin content were observed. Thus, *Volvariella volvacea* polysaccharides hold promise as effective moisturizing, anti-wrinkle and whitening agents in cosmetic formulations.

The cream’s ingredients included deionized water, Sodium Acrylates/Beheneth-25 Methacrylate Crosspolymer, Hydrogenated Polydecene, Lauryl Glucoside, Hydroxyethyl Acrylate/Sodium Acryloyldimethyl Taurate Copolymer, squalane, polysorbate 60, Citric acid, Isopropyl myristate and various concentrations of *V. volvacea* polysaccharides (0.2%, 0.5%, 1.0%), with Phenoxyethanol included in specific proportions [56].

Additionally, extracts from *V. volvacea* were prepared using hot and sonicated aqueous methods, as well as ethanol maceration, and tested for antioxidant activities (DPPH radical scavenging, metal chelating, lipid peroxidation) and in vitro collagen biosynthesis stimulation. Ultrasound-extracted aqueous samples displayed a total phenolic content of 6.68 mg Gallic Acid Equivalent and polysaccharides of 0.069 mg glutamate, with notable DPPH radical scavenging, lipid peroxidation inhibition and collagen biosynthesis stimulation (146.77 ± 13.20% of the negative control), surpassing the effects of ascorbic acid [57]. Extracts of *V. volvacea* stimulate collagen synthesis and have antioxidant effects in vitro. This research continued by experimenting with the bioavailability of different forms of the extract. An extract from *V. volvacea* was placed in niosomes—vesicles composed of non-ionic surfactant molecules. Physicochemical characterization of the niosomes containing the mushroom extract and the gel containing the niosomes of the extract was conducted. The two formulations exhibited superior chemical stability of the phenolic content relative to the extract. The present study has shown the potential of a niosomes gel with *V. volvacea* extract as a suitable delivery system for anti-aging application, due to the improved chemical stability and absorption of the mushroom extract through the rat skin [58].

### 3.3. Preclinical and Clinical Testing of Macrofungal Extracts for Anti-Aging Activity

To introduce macrofungal extracts with anti-aging properties as ingredients in cosmetic and cosmeceutical products, a comprehensive series of preclinical and clinical tests must be conducted to ensure both safety and efficacy before these products reach consumers. The process begins with the assessment of active ingredients, which involves detailed chemical analysis to identify and quantify bioactive compounds such as polysaccharides, polyphenols and terpenoids within the fungal extracts [11,24,31,40,46,50]. This characterization is essential for understanding the components responsible for the anti-aging effects.

Following this, safety evaluation is crucial and involves toxicological testing to assess the extracts’ toxicity. This starts with in vitro studies on various cell lines to determine cytotoxicity, followed by in vivo studies using animal models to evaluate systemic toxicity and establish safe dosage levels [39,49,56]. For example, rodent models may be employed for acute and chronic toxicity tests [18,22,45,51]. Additionally, irritation and sensitization testing is performed to assess potential skin irritation and allergic reactions using standardized dermatological assays [19,21,27,59].

Stability testing is another critical aspect, focusing on the extracts’ viability in cosmetic formulations. This includes evaluating the stability of the extracts under various storage conditions such as temperature, light and humidity, as well as within different cosmetic formulations over time. These stability studies typically involve accelerated aging tests and real-time stability assessments to monitor changes in bioactivity and safety throughout the product’s shelf life. Concurrently, physicochemical properties are examined to ensure compatibility with cosmetic products, which involves analyzing factors such as solubility, pH and viscosity to ensure the extracts remain stable and effective within the formulations [58].

In the clinical phase, the focus shifts to safety evaluation through dermatological trials with human volunteers. Typically, a minimum of 30–50 participants is recommended for initial safety studies to identify any adverse reactions, such as skin irritation or allergic responses. These trials are conducted in controlled environments to ensure accurate and reliable results. Following safety assessments, efficacy testing is conducted with larger groups, often ranging from 100 to 300 participants, to evaluate the extracts’ effectiveness in reducing signs of skin aging [19,56]. Clinical trials measure outcomes such as wrinkle reduction, improvement in skin elasticity and enhanced hydration. Placebo-controlled, double-blind studies are considered the gold standard in this phase to ensure unbiased results and a precise assessment of the extracts’ impact Long-term evaluation is essential to monitor the prolonged effects of the extracts, with studies usually extending over several months to a year to assess sustained safety and efficacy. Participants are observed for any long-term adverse effects and overall improvements in skin condition [21,50].

Finally, regulatory compliance is critical. The final product must adhere to local and international cosmetic and cosmeceutical regulations, requiring thorough documentation of all preclinical and clinical test results and obtaining necessary approvals and certifications from regulatory bodies such as the FDA (U.S. Food and Drug Administration) or EMA (European Medicines Agency). Ensuring adherence to these regulations is essential for confirming that the product meets safety and efficacy standards before it is brought to market.

Conducting these rigorous preclinical and clinical tests is indispensable for validating both the efficacy and safety of macrofungal extracts in cosmetic and cosmeceutical applications, thereby ensuring that they are effective and safe for consumer use.

## 4. Conclusions

**Figure 4:**
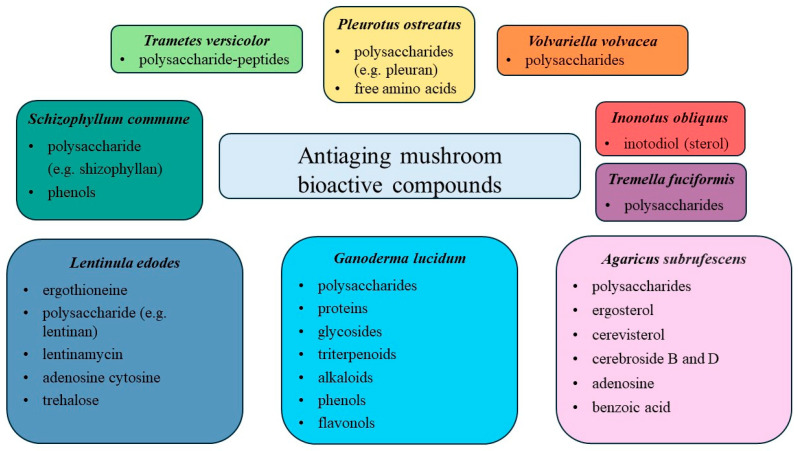
Anti-aging mushroom bioactive compounds for skin.

Numerous experiments unequivocally demonstrate that macroscopic fungi are a valuable source of bioactive compounds with anti-aging potential, making them promising ingredients for anti-aging cosmetics (Figure 4) [13,22,28,48]. Conducted studies confirm that mushrooms contain bioactive compounds such as polysaccharides, terpenoids, polyphenols and peptides, which show a range of beneficial effects on the skin. The polysaccharides in mushrooms, such as beta-glucans, have a moisturizing effect, improving skin elasticity and stimulating collagen production, translating into wrinkle reduction and improving overall skin condition [11,16,43]. Terpenoids exhibit robust antioxidant properties by neutralizing free radicals and shielding the skin from oxidative stress, which is a significant factor in skin aging [47]. Additionally, phenolic compounds found in mushrooms can inhibit the activity of enzymes that break down collagen and elastin, thereby contributing to the maintenance of skin firmness and elasticity [40,44]. Mushroom-derived peptides stimulate skin cell regeneration, improving skin texture and integrity [11,13,30].

The combination of these bioactive compounds makes macroscopic fungi an effective component of anti-aging cosmetic formulations, providing multidimensional benefits for skin health and appearance. Despite the extensive scientific evidence available, additional clinical studies are necessary to more accurately assess the efficacy and mechanisms of action of these ingredients in cosmetic formulations.

In conclusion, the addition of macrofungal extracts in anti-aging cosmetics not only responds to the growing demand for natural and effective skincare products but also opens up new opportunities for innovation in the cosmetic industry. Increasing consumer awareness of the benefits of natural ingredients further highlights the potential of mushrooms as future ingredients in anti-aging cosmetics [Table 1]. There is a need for further research on the use of mushroom extracts in skin applications to mitigate the effects of aging. Based on the analysis of available scientific publications, it can be concluded that more human clinical studies are necessary to confirm the efficacy of formulations containing extracts from mushroom fruiting bodies. Another important aspect of further research on the use of mushroom extracts in cosmetics is the investigation of safety, including the penetration of phenolic compounds into the bloodstream and the frequency of allergic reactions.

## Figures and Tables

**Table 1:** Overview of research on the anti-aging effects of mushroom extracts on skin.

Species	Experiment	Results	
*Trametes versicolor*	in vitro (human keratinocyte HaCaT)	The mushroom extract (10 µg/mL) mitigated UVB-induced cellular senescence in human HaCaT keratinocytes, likely by enhancing the expression of proteins involved in regulating cellular aging, metabolism and stress responses. The extract from *Trametes versicolor* effectively countered UVB-induced cellular senescence through a mechanism that does not depend on reactive oxygen species (ROS).	[12]
	in vitro (human keratinocyte HaCaT)	The enzymatic hydrolysis of polysaccharopeptides from *Trametes versicolor* using 80 U/mL of β-1,3-D-glucanase preserved the functional groups of the polysaccharopeptides while significantly enhancing their antioxidant properties. The resulting modified polysaccharopeptides demonstrated superior antioxidant and anti-inflammatory activities compared to the original polysaccharopeptides.	[13]
*Tremella fuciformis*	in vitro (human skin fibroblasts)	Treatment with hydrogen peroxide led to decreased viability of human skin fibroblasts, accompanied by increased generation of reactive oxygen species and cell apoptosis. However, applying Tremella fuciformis polysaccharide (TFPS) at concentrations ranging from 0 to 400 µg/mL for up to 48 h did not affect cell viability. Pre-treatment with the polysaccharide effectively mitigated oxidative stress and reduced apoptosis in hydrogen peroxide-treated fibroblasts. The protective effect of the polysaccharide was concentration-dependent, with the maximum effect observed at 200 µg/mL. These findings suggest that TFPS alleviates hydrogen peroxide-induced oxidative stress and apoptosis in skin fibroblasts by upregulating proteins involved in cellular aging, metabolism and stress responses.	[14]
	in vitro (human keratinocyte HaCaT)	Extracts of *Tremella fuciformis* at concentrations of 100, 200 and 300 µg/mL significantly reduced melanin levels and tyrosinase expression in a dose-dependent manner in murine B16F10 cells. Additionally, concentrations of 100 and 200 µg/mL of *Tremella fuciformis* extract enhanced wound healing in human keratinocytes and Detroit 551 fibroblasts. These findings demonstrate the extract’s effective inhibition of melanogenesis and its promotion of wound healing in vitro. Notably, the treatment did not alter cell morphology or significantly impact the viability of B16F10 cells.	[15]
	in vitro (human skin fibroblasts)	Pretreatment with Tremella fuciformis polysaccharides (3.125–400 mg/mL) effectively mitigated oxidative stress in UVA-exposed human dermal fibroblasts. This treatment significantly reduced the levels of reactive oxygen species and malondialdehyde while enhancing total antioxidant activity. Key antioxidant enzymes, including catalase, superoxide dismutase and glutathione peroxidase, showed marked increases. Additionally, polysaccharide pretreatment protected fibroblasts by upregulating the protective protein Nrf2 and downregulating Keap1 expression. The polysaccharides also increased collagen I, elastin and hyaluronic acid levels in UVA-treated skin fibroblasts.	[16]
	in vitro (human keratinocyte HaCaT)	A range of studies was conducted to assess the effects of polysaccharides on UV-damaged human skin in a chronic UV-irradiated mouse model. The research determined that polysaccharides at a concentration of 5 mg/mL effectively protected human skin keratinocytes from UV-induced apoptosis and reactive oxygen species production. They modulated the levels of thioredoxin-interacting protein and thioredoxin reductase 2, contributing to photoprotection. Additionally, topical application of polysaccharides alleviated UV-induced skin damage in the chronic UV-irradiated mouse model. The assays indicated that concentrations of 1–5 mg/mL of *Tremella fuciformis* polysaccharides (TFPS) were safe for human skin keratinocytes and significantly enhanced cell viability	[17]
	in vivo (six-week-old female Babl/c mice)	In this study, polysaccharides extracted from *Tremella fuciformis* using hot water extraction and ethanol precipitation were evaluated for their therapeutic effects. The efficacy of topical versus oral administration of these polysaccharides was compared in a dinitrofluorobenzene-induced atopic dermatitis mouse model. Both routes of administration (50 and 200 mg/kg) improved transdermal water loss, epidermal thickening and ear edema in the atopic dermatitis mice. However, oral administration demonstrated significantly superior efficacy compared to topical application. Additionally, polysaccharide treatment led to an increased proportion of regulatory T cells in the mesenteric lymph nodes.	[18]
	human clinical studies (20 Thai volunteers)	Stable hand sanitizer gel bases were developed with 66.5% ethanol and 0.3% triclosan, and were enhanced with polysaccharides extracted from snow mushrooms. Among the formulations tested, those containing 10% snow mushroom extract and 0.3% gelling agent were preferred most by 20 Thai volunteers. The snow mushroom hand sanitizer was found to be non-irritating, similar to the placebo. Additionally, the snow mushroom gel significantly improved skin moisture compared to the placebo at all time points, up to the end of the 180-min study period (*p* < 0.05).	[19]
	human clinical studies	Cosmetic formulations incorporating *Tremella fuciformis* extract demonstrated a 12.4% reduction in transepidermal water loss compared to formulations lacking this active ingredient, effectively maintaining epidermal hydration as confirmed by instrumental measurements. The emulsion base comprised the following components: Citric Acid (for pH regulation), Sodium Gluconate (0.2%), Xanthan Gum (1.0%), Sodium Benzoate + Potassium Sorbate + Aqua (1.0%), Tocopherol + *Helianthus annuus* Seed Oil (1.0%), Methyl Glucose Sesquistearate (2.0%), Glycerin (7.0%), *Vitis vinifera* (Grape) Seed Oil (25.0%) and Aqua (to 100.0%). *Tremella* fungus extract (Evonik) was incorporated into the emulsion at a concentration of 0.1 wt.%. Dermatological tests performed on the final formulations revealed no instances of erythema, swelling or irritation in any of the test subjects 48 and 72 h post-application.	[21]
	in vitro (chemical determination of radical scavenging activity by 3 methods) and in vivo (Specific-pathogen-free (SPF) grade 4-week-old male Kunming mice)	Purified polysaccharides from *Tremella fuciformis* (molecular weight 4.68 × 10^6^ Da), composed of mannose, fucose, xylose and glucose, were evaluated for their biological activity. These polysaccharides lacked a triple helix conformation and demonstrated scavenging activity against radicals that was dependent on both concentration and molecular weight. Notably, the polysaccharides significantly mitigated skin aging, reduced oxidative stress and diminished inflammation in a model of d-galactose-induced aging in mice.	[22]
*Ganoderma lucidum*	in vitro (DPPH, FRAP tyrosinase inhibition and B16 melanoma cells)	Five varieties of *Ganoderma lucidum* from Vietnam were analyzed for their total polyphenol content and antioxidant properties, including DPPH radical scavenging, FRAP assay and tyrosinase inhibition. All extracts demonstrated notable bioactivity, which correlated strongly with their total polyphenol content, with Pearson correlation coefficients approximately r ~ 0.8 (*p* < 0.10). The ethanol extract of Korean lingzhi, noted for its high bioactivity, was characterized by the presence of 16 bioactive compounds, including proteins, glucosides, triterpenoids, alkaloids, phenolic compounds, flavonoids and polysaccharides. At a concentration of 40 μg/mL, this extract was tested on B16 melanoma cells and showed no significant cytotoxicity. It effectively inhibited melanin production by 29.34% and reduced intracellular tyrosinase activity by 21.93%, comparable to the positive control, arbutin (*p* > 0.05). These findings suggest that *Ganoderma lucidum* holds substantial promise for use in skincare products aimed at treating skin pigmentation.	[24]
	in vitro (inhibition of tyrosinase)	The inhibitory effects on tyrosinase activity were assessed for extracts from various mushrooms, including *Ganoderma lucidum*, *Antrodia camphorata*, *Agaricus brasiliensis* and *Cordyceps militaris*. Among these, *Ganoderma lucidum* demonstrated the most significant inhibition of tyrosinase activity, with an IC50 value of 0.32 mg/mL. At a concentration of 1 mg/mL, *Ganoderma lucidum* extracts reduced tyrosinase activity by approximately 80%. In contrast, a concentration of 0.1 mg/mL resulted in a 40% reduction in tyrosinase activity.	[25]
	in vitro (mouse melanoma cell line, B1610F7)	Methanolic extracts of *Ganoderma lucidum* demonstrated an inhibitory effect on melanin biosynthesis in the B16F10 mouse melanoma cell line. An active compound, ergosterol peroxide, was isolated from the extract and found to significantly reduce melanin accumulation at a concentration of 2 mg/mL. This reduction was attributed to the suppression of melanogenic enzymes in the B16F10 cells, with greater efficacy observed at concentrations exceeding 1 mg/mL.	[26]
	in vitro (peripheral blood mononuclear cells (PBMCs))	A method was employed to extract β-glucan from the antler-type fruiting body of *Ganoderma lucidum*. The resulting extract, with a concentration of 0.5 mg/mL, contained 40.57% β-glucan and 7.47% protein. This extract exhibited notable bioactivities, including anti-tyrosinase and antioxidant properties, making it a potential agent for skin whitening by reducing or inhibiting pigmentation processes. Additionally, the β-glucan demonstrated moderate activities against collagenase, elastase and hyaluronidase. The extract was found to be non-irritating to the skin, and tests with human peripheral blood mononuclear cells (PBMCs) indicated no significant impact on cell viability.	[27]
	in vitro (B16F10 and PIG1 cells)	The expression of melanogenesis-related genes, including microphthalmia-associated transcription factor, tyrosinase, tyrosinase-related protein 1, tyrosinase-related protein 2, ras-related protein Rab-27A and myosin, increases following UVB irradiation in B16F10 and PIG1 cells. However, treatment with *Ganoderma lucidum* polysaccharide (40 µg/mL) effectively downregulates these UVB-induced melanogenesis genes. The polysaccharides inhibit UVB-induced activation of protein kinase A and mitogen-activated protein kinase signaling pathways, protect mitochondria from UVB-induced damage and reduce reactive oxygen species production. In zebrafish models of UVB-induced skin pigmentation, the polysaccharides demonstrated the ability to suppress UVB-induced pigmentation. Additionally, in guinea pig models, the polysaccharides significantly alleviated erythema caused by high-dose UVB exposure. Notably, low concentrations of *Ganoderma* polysaccharides (<160 µg/mL) did not exhibit significant toxicity towards B16F10 cells.	[28]
	in vitro (human skin fibroblasts)	Six polysaccharides from *Ganoderma lucidum* were assessed for their free radical scavenging abilities (DPPH, ABTS, hydroxyl and superoxide anion radicals) and their effects on oxidative damage induced by hydrogen peroxide (H_2_O_2_) in human skin fibroblasts. One polysaccharide, GLP1, was further fractionated into GLP1I and GLP1II. All polysaccharides demonstrated effective free radical scavenging and enhanced fibroblast resistance to H_2_O_2_ damage. At a concentration of 5 g/L, GLP1, GLP1I and GLP1II significantly reduced reactive oxygen species (ROS) and malondialdehyde (MDA) levels. GLP1II was notably more effective than vitamin C in protecting cells. Additionally, these polysaccharides increased the activities of superoxide dismutase (SOD), catalase and glutathione peroxidase (GPx). The polysaccharides also influenced signaling pathways by activating key antioxidant genes and inhibiting negative regulators. Overall, GLP1, GLP1I and GLP1II effectively protected human skin fibroblasts from H_2_O_2_-induced oxidative damage, highlighting their potential as natural antioxidants for skin protection.	[29]
	in vitro *Mus musculus* skin melanoma cell line (B16-F10 ATCC, CRL-6475) and Cercopithecus aethiops kidney normal cell line (Vero ATCC, CCL-81); mouse fibroblast cell line (L929)	The multifunctional peptide derived from *Ganoderma lucidum* was sequenced via LC-MS/MS as NH2-PVRSSNCA-CO2H (octapeptide). Its antioxidant activity was evaluated at 1 mg/mL using DPPH, ABTS and FRAP assays. The octapeptide demonstrated antioxidant capacities of 0.121 ± 0.01 mg ascorbic acid equivalent, 0.173 ± 0.03 mg gallic acid equivalent and 2.21 ± 0.23 mM FeSO_4_ equivalent, comparable to established antioxidants. Proteomics analysis identified 5804 proteins and highlighted several pathways affected by the octapeptide in melanoma cells. Targeted proteomics revealed that pigmentation-related proteins were upregulated, while Tyrosinase-Related Protein 1 was downregulated in the treated group. The octapeptide, at concentrations from 1.5625 µg/mL to 100 µg/mL, did not cause significant cell death in either melanoma or Vero cells.	[30]
*Lentinula edodes*	in silico	Three bioactive compounds were isolated from *Lentinula edodes*, with compound 1 previously reported and compounds 2 and 3 newly identified. The study indicates that compounds 1 and 3 contribute to the fruit body’s potential as an effective cosmetic ingredient for skin lightening or treating melasma. Notably, compounds 2 and 3 are being reported from *Lentinula edodes* for the first time. Compound 3, in particular, shows promise as a lead molecule for targeting tyrosinase, with potential for optimization to enhance efficacy and reduce toxicity. This compound also exhibits significant skin-lightening effects by inhibiting melanin production.	[32]
	in vitro (human keratinocyte HaCaT)	Lentinan extracted from *Lentinula edodes* exhibited significant antioxidant properties, effectively quenching DPPH (0.094–1.5 mg/mL), ABTS (23.44–375 µg/mL) and superoxide anions (23.44–375 µg/mL) in a concentration-dependent manner. It reduced malondialdehyde formation and increased superoxide dismutase activity. Lentinan not only provided protection against oxidative damage but also demonstrated reparative effects in keratinocyte cells, enhancing cellular tolerance to oxidative stress and improving cell repair mechanisms.	[34]
	in vitro (Human malignant melanoma cell line (A375.S2) and human foreskin fibroblast (HS27)) In vivo (Zebrafish embryos (Danio rerio))	This study evaluates the effects of mutated Shiitake extract (A37) and wildtype Shiitake extract (WE) on various activities. Although both extracts have similar total phenolic contents, A37 has a higher total flavonoid content (1.04 ± 0.7 mg/100 mL) compared to WE (0.86 ± 0.9 mg/100 mL). A37 showed lower antioxidant activity (EC50 = 549.6 ± 2.70 µg/mL) than WE (EC50 = 52.8 ± 1.19 µg/mL). Toxicity tests on zebrafish embryos indicated that both extracts halted embryogenesis at concentrations above 900 µg/mL. Both extracts reduced pigmentation, but A37 did not affect embryo heartbeat. Cell cycle analysis revealed that WE significantly altered the cell cycle, whereas A37 did not. Both extracts inhibited phosphorylation of Glycogen Synthase Kinase 3 β in human foreskin fibroblasts, potentially triggering apoptosis in melanin-producing cells. Of 19 known compounds, 14 were present in both extracts, with decitabine in A37 highlighting its potential for medical applications such as melanoma treatment and skin therapy.	[39]
	in vitro (total phenolic content (TPC), antioxidant activities—DPPH ABTS)	The results indicated that *V. volvacea* exhibited the highest protein content (28.70%) and ash content (9.48%). Significant differences were observed between some tray-dried and freeze-dried mushrooms. Tray drying generally enhanced the total phenolic content, as well as DPPH and ABTS antioxidant activities in *P. ostreatus*, *P. pulmonarius* and *L. edodes*. Among the five mushroom species studied, both methanol and hot water extractions of 10 g of *V. volvacea* yielded the highest total phenolic content and antioxidant values for DPPH and ABTS, with no significant differences between tray-dried and freeze-dried methods. Additionally, hot water extracts provided higher yields of gallic acid and p-hydroxybenzoic acid compared to methanol extracts. Consequently, tray-dried *V. volvacea* demonstrated the greatest potential as a natural antioxidant.	[40]
*Schizophyllum commune*	in vitro (ABTS, DPPH mouse B16F10 cells)	In this study, *S. commune* strains from various provinces in China were screened for schizophyllan production. The strain NTU-1, which produced a high yield of schizophyllan, was selected for further evaluation. The bioactivity of schizophyllan from NTU-1 was assessed, revealing its potential for skincare due to its antioxidant, anti-photoaging and melanin-inhibiting properties. The optimal concentration for antioxidant activity was found to be 2.0 mg/mL, while the maximum UVB protection was achieved at 1 mg/mL.	[41]
	in vitro (DPPH, FRAP)	Antioxidant properties of hot water extract (HWE), hot water-extracted polysaccharides (HWP) and hot alkali-extracted polysaccharides (HWAE) from the fruiting bodies of *Schizophyllum commune* were analyzed. All extracts contained both α- and β-glucans, with glucose as the predominant monosaccharide. The total phenol content decreased in the order HWP ≈ HWE > HWAE. The median effective concentrations (EC50) for antioxidant activities were 8.3 ± 0.1 mg/mL for HWE, 6.9 ± 0.0 mg/mL for HWP and 8.9 ± 0.1 mg/mL for HWAE. For DPPH scavenging activity, EC50 values were 0.8 ± 0.0 mg/mL (HWE), 0.6 ± 0.0 mg/mL (HWP) and 1.8 ± 0.0 mg/mL (HWAE). Reducing power EC50 values were 7.6 ± 0.1 mg/mL (HWE), 7.9 ± 0.0 mg/mL (HWP) and 12.5 ± 0.1 mg/mL (HWAE). For ferrous ion chelation, EC50 values were 3.1 ± 0.0 mg/mL (HWE), 4.6 ± 0.1 mg/mL (HWP) and 4.9 ± 0.1 mg/mL (HWAE). The EC50 values for antioxidant activity, DPPH scavenging and reducing power were positively correlated with both total polysaccharide and total phenol content.	[42]
	in vitro DPPH, FRAP, ABTS, human skin fibroblasts (HSF)	This study assessed the antioxidant activities of two polysaccharides, intracellular polysaccharides (IPS) and extracellular polysaccharides (EPS), at biochemical, cellular and molecular levels using an H_2_O_2_-induced oxidative damage model in fibroblasts. Both IPS and EPS demonstrated significant antioxidant effects, improving cellular levels of superoxide dismutase (SOD) and reducing malondialdehyde (MDA). Molecular analysis revealed that both polysaccharides enhanced the expression of a transcription factor that regulates antioxidant genes while inhibiting the expression of a negative feedback gene. The optimal concentrations of IPS and EPS were determined to be 2.5 mg/mL.	[43]
	in vitro (DPPH, FRAP, Scavenging effect on superoxide SOA, tyrosinase inhibition activity)	The study found that extracting *Schizophyllum commune* at 4 °C or 30 °C for 1 h yielded extracts (10 mg/mL) with the highest anti-pigmentation effects, achieving 94.2% and 95.4% inhibition, respectively. At 4 °C, shorter extraction times were more effective for ferric-reducing and DPPH-radical scavenging activities, while results at 30 °C varied. Therefore, the optimal conditions for obtaining effective cosmeceutical properties from *S. commune* are 30 °C extraction for 1 h.	[44]
	in vitro (the abdominal skin of female hairless guinea pigs (strain IAF/HA-hrBR)) in vivo (New Zealand albino rabbit)	This study aimed to evaluate the impact of papain, a proteolytic enzyme, on drug percutaneous absorption. To ensure enzyme stability during skin penetration, papain was conjugated with glucan. The *Schizophyllum commune* glucan-papain conjugate significantly enhanced the percutaneous absorption of antipyrine. Microscopic analysis revealed an increase in the thickness of the stratum corneum and viable epidermis following treatment with the conjugate. These structural changes are likely due to the hydrolysis of extensive cross-linking in corneocyte envelopes and intracellular proteins. Importantly, the *S. commune* glucan-papain conjugate did not cause skin irritation according to the Draize test. The cumulative absorption of antipyrine over 10 h was approximately eleven times higher compared to the control when skin was pre-treated with a 2.0% conjugate solution.	[59]
*Inonotus obliquus*	in vitro (human dermal fibroblasts) in vivo (female SKH-1 hairless mice)	Pretreatment with *Inonotus obliquus* effectively scavenged intracellular reactive oxygen species and prevented lipid peroxidation in hydrogen peroxide-treated human fibroblasts. The ROS scavenging activity of *I. obliquus* was 33% at a concentration of 25 µg/mL, compared to 7% inhibition in untreated cells. Additionally, *I. obliquus* exhibited protective effects against hydrogen peroxide-induced apoptosis and premature senescence in fibroblasts. In vivo, *I. obliquus* also mitigated UV-induced skin changes, including thickening and wrinkle formation, in hairless mice and enhanced collagen synthesis by inhibiting metalloproteinase activity. Control cell viability decreased to 48.4% following hydrogen peroxide treatment, while treatment with *I. obliquus* at 25 µg/mL restored cell viability to 69.5%.	[45]
	in vitro (human dermal fibroblast cells HDF)	Double-strand RNA (dsRNA), which can trigger inflammation, is generated by necrotic keratinocytes in the skin. Inotodiol, a natural lanostane-type triterpenoid isolated from *Inonotus obliquus* (Chaga mushroom), is known for its significant pharmacological and anti-inflammatory properties. This study evaluated the anti-inflammatory effects of inotodiol on poly(I:C)-induced inflammation in fibroblasts. The results demonstrated that inotodiol effectively mitigates inflammation induced by poly(I:C) in fibroblasts. Poly(I:C) at concentrations ranging from 0 to 80 µg/mL did not cause cytotoxicity, while inotodiol showed approximately 70% cytotoxicity at concentrations of 20 µg/mL or higher.	[46]
	in vitro (human keratinocyte HaCaT)	A lanostane triterpenoid-rich concentrate from *Inonotus obliquus* (Chaga), containing 10% inotodiol, was compared with pure inotodiol for anti-inflammatory effects on a human keratinocyte cell line. Both were tested under various conditions, including UVB irradiation and tumor necrosis factor. Pure inotodiol effectively suppressed interleukin-induced inflammation at 0.44–4.0 μg/mL. UVB-induced pro-inflammatory cytokines were significantly reduced by both pure inotodiol and the inotodiol concentrate. The concentrate also modulated collagen and hyaluronic acid synthesis, with decreased mRNA expression of cytokines at 2.5 µg/mL. Inotodiol was non-toxic at concentrations up to 20 µg/mL, with cell viability above 70%.	[47]
*Pleurotus ostreatus*	in vitro (DPPH, ABTS, Hs68—Human Foreskin Fibroblast cell line)	This study examined the effects of *Pleurotus ostreatus* polysaccharides (POP) on fibroblasts. POP-40, POP-60 and POP-80 were extracted using 40%, 60% and 80% ethanol gradient precipitation, respectively. The results demonstrated that these polysaccharides exhibited significant DPPH and ABTS radical scavenging activities, water-retention capacity and inhibition of collagenase and elastase, with POP-80 showing the highest efficacy. Following UVA irradiation, fibroblasts exhibited increased reactive oxygen species, senescent cells and pro-inflammatory cytokines. However, pretreatment with 50 μg/mL of polysaccharides notably reduced reactive oxygen species and the number of senescent cells, decreased NF-κB activity and inhibited interleukin-6.	[48]
	in vitro (human keratinocyte HaCaT and fibroblast (HFF-1) cell lines)	In vitro safety assessments of the extracts and cosmetic formulations were conducted using keratinocyte and fibroblast cell lines. The results indicated that the extracts did not exhibit toxicity to either cell type, demonstrating their safety for use in cosmeceuticals. Protocatechuic and syringic acids were the only compounds from *Ganoderma lucidum* extract that penetrated within the first 8 h, while phenolic acids from *Pleurotus ostreatus* extract showed no penetration. In HaCaT cells, exposure to the extracts maintained up to 90% cell viability at 100 μg/mL. However, at the highest concentration tested (10 mg/mL), a significant reduction in cell viability was observed.	[49]
	human clinical studies (face and body in 20 human subjects with all skin types) (sensitive, atopic and normal) and phototype II and III)	This study aimed to assess the soothing effects of a β-glucan pleuran-based cream on skin damage induced by UV exposure (UVA/UVB). The cream significantly reduced erythema compared to the control. Over a 30-day period, it improved various skin parameters, including moisture, brightness, elasticity and total antioxidant capacity. The cream formulation comprised the following ingredients: aqua (72.0%), glycerin (5.0%), *Helianthus annuus* seed oil (7.0%), stearic acid (3.0%), cetyl alcohol (3.0%), β-glucan (from *Pleurotus ostreatus*, 2.0%), sorbitan stearate (1.0%), polysorbate 60 (1.0%), phenoxyethanol (1.0%), tocopherol (0.5%), allantoin (0.5%), xanthan gum (0.5%) and citric acid (0.5%).	[50]
	in vivo (66 female mice)	This study investigated the wound-healing properties of topical *Pleurotus ostreatus* extract in albino mice using an excisional wound model. The extract, formulated with Vaseline at 5% and 10% concentrations, significantly enhanced wound closure and improved histopathological and immunohistochemical parameters. The 10% (*w*/*w*) concentration was found to be safe and effective, with no adverse effects observed over a 14-day period.	[51]
	in vitro	Free amino acids, key components of the natural moisturizing factor, are crucial for maintaining skin hydration, and their deficiency can lead to dry skin conditions. This study aimed to analyze the free amino acid content in selected Ethiopian plant and fungal species, specifically *Pleurotus ostreatus*. The extract of *Pleurotus ostreatus* was found to contain a high concentration of free amino acids, with a total content of 400.01 mg/g.	[52]
*Agaricus blazei*	in vitro (cell line B16F10 from skin tissue of a mouse with melanoma; human dermal fibroblast; RAW 264.7 cell—Macrophage from blood; keratinocytes HaCaT)	The extract of *Agaricus blazei* demonstrated inhibition of melanin synthesis, enhanced collagen production and upregulated the expression of hyaluronan synthase-2, hyaluronan synthase-3 and aquaporin-3 at a concentration of 100 µg/mL. The identified components of the extract include ergosterol (1), 5-dihydroergosterol (2), cerevisterol (3), cerebroside B (4), cerebroside D (5), adenosine (6) and benzoic acid (7). Among these, 5-dihydroergosterol (2) inhibited melanogenesis in B16F10 cells and promoted collagen synthesis in human dermal fibroblasts. Additionally, cerevisterol (3), cerebroside B (4) and cerebroside D (5) reduced nitric oxide production in RAW 264.7 cells. Notably, cerebroside D (5) increased the expression of hyaluronan synthase-2 and aquaporin-3 genes in keratinocytes.	[53]
	in vitro (normal human epidermal melanocytes (NHEM); Murine monocyte/macrophage RAW264.7 cells culture)	The effects of *Agaricus blazei* on tyrosinase activity were assessed using L-tyrosine and L-DOPA in normal human epidermal melanocytes. The extract inhibited tyrosinase activity in a dose-dependent manner, similar to arbutin and Vitamin C. Treatment with *Agaricus blazei* extract (3–100 µg/mL) reduced melanin content by up to 57% compared to the control. The extract also suppressed NO production in melanocytes and in LPS-stimulated RAW264.7 macrophages, without affecting iNOS mRNA expression. These findings suggest that *Agaricus blazei* extract inhibits melanin production by partially inhibiting tyrosinase activity and reducing NO production.	[54]
	in vitro (B16-F10 mouse melanoma cell line)	The methanol extract of *Agaricus brasiliensis* demonstrated significant inhibition of melanin synthesis and reduced expression of melanogenesis-related proteins. It decreased intracellular reactive oxygen species levels and inhibited mushroom tyrosinase activity. The extract showed an IC50 of 0.713 mg/mL for intracellular tyrosinase activity and an IC50 of 0.711 mg/mL for melanin reduction in B16F10 cells. Additionally, it lowered the protein expression of tyrosinase and tyrosinase-related protein 1. These results indicate that *Agaricus brasiliensis* methanol extract effectively inhibits melanogenesis and reduces intracellular reactive oxygen species in B16F10 cells.	[55]
*Volvariella volvacea*	in vitro (Human dermal skin fibroblast cells) Human clinical studies (20 healthy volunteers with no historical skin allergy)	Polysaccharides from *Volvariella volvacea* were evaluated for cosmetic applications and in vivo efficacy. Three extraction methods—hot water shaking (HS), microwave-assisted (MA) and ultrasonic-assisted (UA)—were compared. HS yielded the highest extraction rate (15.58 ± 0.96% *w*/*w*) and beta-glucan content (18.80 ± 0.81% *w*/*w*). HS polysaccharides demonstrated the strongest inhibition of lipid peroxidation (IC50 = 0.0378 mg/mL), tyrosinase (51.46 mg KAE/g) and elastase (604.21 ± 73.66 mg EGCG/g). The polysaccharides showed no cytotoxicity to human dermal fibroblasts. The cream containing these polysaccharides, though offering lower sun protection factor, significantly enhanced skin moisture, gross elasticity, net elasticity and firmness. The cream formulation included deionized water, sodium acrylates/beheneth-25 methacrylate crosspolymer, hydrogenated polydecene, lauryl glucoside, hydroxyethyl acrylate/sodium acryloyldimethyl taurate copolymer, squalane, polysorbate 60, citric acid, isopropyl myristate, *V. volvacea* polysaccharides (0.2%, 0.5%, 1.0%) and phenoxyethanol.	[56]
	in vitro (the human fibroblasts)	Eleven mushrooms were extracted using three methods: hot aqueous (HW), sonicated aqueous (SW) and macerated ethanolic (ME). The sonicated aqueous extract of *Volvariella volvacea* (VV SW) exhibited the highest total phenolic content (6.68 mg GAE) and polysaccharide content (0.069 mg GLU). It also demonstrated the greatest DPPH radical scavenging activity, lipid peroxidation inhibition and collagen biosynthesis stimulation, with collagen biosynthesis at 146.77 ± 13.20% of the negative control, significantly surpassing ascorbic acid by approximately 1.14 times.	[57]
	in vitro (abdominal skin the male Sprague–Dawley rats)	This study investigated the incorporation of *Volvariella volvacea* extract into niosomes and assessed the physicochemical properties of these niosomes and the gel containing them. Transdermal absorption through rat skin was evaluated using Franz diffusion cells over 6 h, comparing niosome-loaded extract with a solution of the extract. Niosomes demonstrated greater chemical stability for total phenolic contents compared to the extract solution. They had a mean size of 254 ± 20.32 nm and exhibited a negative zeta potential. While niosomes initially reduced the cumulative and flux amounts of total phenolics in the first hour, they significantly enhanced skin permeation by the sixth hour. Overall, niosomes achieved the highest percentage of total phenolic content permeation through rat skin.	[58]

## Data Availability

Extracted data used in the review are available upon reasonable request.
